# Cost-utility of first-line dostarlimab plus carboplatin-paclitaxel compared with placebo for patients with pA/rEC: a partitioned survival analysis

**DOI:** 10.1186/s13561-026-00778-0

**Published:** 2026-04-23

**Authors:** Ziyad S. Almalki, Saba S. Albanna, Yahia A. Hadadi, Ahmed M. Alshehri, Kamar Z. Jamal, Saja H. Almazrou, Jameilh A. Alsamiri, Abdulrahman A. Alsuhibani, Abdullah A. Alalwan, Ahmad A. Alamer, Nehad J. Ahmed, Abeer A. Shhada, Ghuafran A. Alnajem

**Affiliations:** 1 Department of Clinical Pharmacy, College of Pharmacy, Prince Sattam Bin Abdulaziz University, Al-Kharj, Riyadh, Saudi Arabia; 2 Department of Clinical Pharmacy, College of Pharmacy, AlMaarefa University, Riyadh, Saudi Arabia; 3 Department of Sales, A. Menarini Farmaceutica Internazionale S.R.L, Riyadh, Saudi Arabia; 4 Department of Clinical Pharmacy, College of Pharmacy, King Saud University, Riyadh, Saudi Arabia; 5 Department of Pharmacy Practice, College of Pharmacy, Qassim University, Qassim, Saudi Arabia

## Abstract

**Background:**

Given the rising incidence of endometrial cancer in Saudi Arabia, the integration of high-cost therapeutics, such as dostarlimab (DSR), for primary advanced or recurrent (pA/rEC) disease necessitates rigorous economic appraisal. Therefore, this study aimed to evaluate the cost-utility of first-line dostarlimab plus carboplatin-paclitaxel (DSR + CP) versus placebo plus carboplatin-paclitaxel (PBO + CP) from the perspective of the Saudi healthcare payer.

**Methods:**

A partitioned survival model was constructed to simulate patient prognosis across three health states: progression-free disease, progressive disease, and death. The model parameters were derived from the clinical efficacy data of the RUBY trial and the local cost inputs. The primary outcome was the incremental cost-effectiveness ratio (ICER), the robustness of which was evaluated via comprehensive deterministic and probabilistic sensitivity analyses to characterize parameter uncertainty.

**Results:**

For the overall pA/rEC population, the DSR + CP regimen yielded an incremental 1.55 quality-adjusted life-years (QALYs) at an additional cost of $144,716, resulting in an ICER of $93,244 per QALY. Cost-effectiveness was more favorable in the mismatch repair deficient (dMMR) subgroup (ICER: $82,754/QALY) than in the mismatch repair proficient (pMMR) cohort (ICER: $132,571/QALY). The ICER is the most sensitive to the acquisition cost of the DSR. At a willingness-to-pay threshold of $90,000/QALY, the probability of DSR + CP being cost-effective was 83.9% for the dMMR subgroup.

**Conclusion:**

This economic evaluation indicates that DSR + CP constitutes a cost-effective intervention for pA/rEC within the dMMR subpopulation. These findings provide strong evidence for a tailored reimbursement policy that effectively utilizes resources by connecting clinical benefits with economic value in the management of pA/rEC.

**Supplementary Information:**

The online version contains supplementary material available at 10.1186/s13561-026-00778-0.

## Key points

Given the rising incidence of endometrial cancer and financial pressures on the Saudi healthcare system, this study provides crucial, locally-specific economic evidence to inform policy and reimbursement decisions for the high-cost drug dostarlimab. This analysis reveals that adding dostarlimab to chemotherapy is only cost-effective for a specific subgroup of endometrial cancer patients (dMMR) in Saudi Arabia. Therefore, the findings strongly recommend mandatory biomarker testing to select the right patients for this expensive treatment and suggest that significant price negotiations are necessary to make it a viable option.

**Supplementary Information:**

The online version contains supplementary material available at 10.1186/s13561-026-00778-0.

## Introduction

Endometrial cancer (EC) is a prominent gynecological malignancy and a significant public health issue worldwide [1]. It ranks as the 15th most common cancer and the sixth most common cancer among women, with an estimated 420,368 new cases and 97,723 deaths attributed to it globally by 2022 [2]. In Saudi Arabia (SA), EC is a major national health concern. It is the fourth most common cancer among Saudi females in both 2020 and 2021, with 631 new cases recorded in the latter year, accounting for 5.9% of all female cancers [3]. The age-standardized incidence rate (ASR) in Saudi Arabia was 12.1 per 100,000 in 2021 [4], a figure underscored by a study citing a dramatic 1092.3% increase in EC ASR between 1990 and 2019 [5]. While specific mortality data for EC in the Kingdom are not consistently detailed, the rising overall cancer mortality and number of cases diagnosed at advanced stages suggest that EC contributes meaningfully to the national cancer mortality burden.

Systemic therapy is central to the management of patients with primary advanced or recurrent endometrial cancer (pA/rEC). The established first-line standard of care is platinum-based chemotherapy, typically a combination of carboplatin and paclitaxel (CP) [6]. However, despite the initial responses, the long-term efficacy of this approach is limited, as most patients eventually experience disease progression [7]. This landscape of high recurrence rates underscores the crucial need to optimize initial therapy and achieve more durable responses, thereby prolonging survival and improving the quality of life [8].

The emergence of immune checkpoint inhibitors has marked a significant advancement in this setting. The efficacy and safety of adding the PD-1 inhibitor dostarlimab (DSR) to CP were established in Part 1 of the global, randomized, double-blind, placebo-controlled phase III RUBY trial [9]. This study demonstrated that dostarlimab plus carboplatin-paclitaxel (DSR + CP) provides a substantial clinical benefit compared with placebo plus carboplatin-paclitaxel (PBO + CP), particularly for the subpopulation with mismatch repair-deficient/microsatellite instability-high (dMMR) tumors. A more modest benefit was observed in the mismatch repair proficient/microsatellite stable (pMMR) subgroup. The safety profile of DSR + CP was found to be generally manageable and consistent with the known profiles of individual agents [9].

Although the introduction of DSR for treating pA/rEC is a significant clinical advance, its high cost necessitates careful economic evaluation [10]. A consistent finding across international health economic literature is that the regimen’s value is not uniform but is critically dependent on the tumor’s mismatch repair (MMR) status. The pronounced clinical benefit observed in mismatch repair deficient (dMMR) patients compared to that in pMMR patients is the primary driver of this economic disparity.

International analysis has consistently validated this principle. An analysis from a United States (US) payer perspective found DSR + CP to be cost-effective for the dMMR population, with a favorable Incremental Cost-Effectiveness Ratio (ICER) per Quality-Adjusted Life Year (QALY), as well as for the overall population, although at a higher ICER [10]. A separate US study, using a standard willingness-to-pay (WTP) threshold, also found that the DSR + CP regimen was cost-effective for dMMR pA/rEC, with an ICER well within the acceptable range. However, for the pMMR pA/rEC, the ICER exceeded this threshold, indicating that DSR + CP was not cost-effective [11]. This pattern was mirrored in a Canadian analysis, which, using its threshold, reported a highly cost-effective ICER for the dMMR subgroup, whereas the pMMR subgroup’s ICER was substantially above the threshold; therefore, DSR + CP was not considered cost-effective [12]. Similarly, research from China comparing DSR + CP to PBO + CP yielded a cost-effective ICER for the dMMR subgroup, in contrast to a significantly higher and less favorable ICER for the pMMR subgroup [13].

However, these international findings are not directly transferable to the Saudi Arabian context due to its unique healthcare financing and policy landscape. The Saudi healthcare system is primarily funded by the state, with the Ministry of Health (MOH) offering most services at no cost to patients [14]. However, this system is under considerable long-term financial pressure, highlighted by projections estimating a 116.7% increase in new cancer cases between 2020 and 2040 [15].

This policy evolution highlights a critical evidence gap; there is no published cost-effectiveness analysis of the DSR + CP regimen from a Saudi payer perspective. The lack of locally relevant data limits Saudi healthcare decision-makers’ ability to conduct informed value assessments, negotiate prices effectively, and optimally allocate resources to improve population health.

Accordingly, the primary objective of this study was to evaluate the cost-effectiveness of DSR + CP compared to PBO + CP for the first-line treatment of pA/rEC from the perspective of the Saudi healthcare payer. By quantifying the incremental costs and health benefits, this research will provide crucial local evidence needed to inform healthcare policies, reimbursement decisions, and clinical practice in Saudi Arabia.

## Methods

### Study design

This study evaluated the cost-utility of DOS plus carboplatin-paclitaxel compared with PBO + CP as a first-line treatment for pA/rEC. The analysis was performed from the Saudi payer perspective, and its methodology and reporting adhered to the Consolidated Health Economic Evaluation Reporting Standards (CHEERS) [16].

### Model structure

**Fig. 1:**
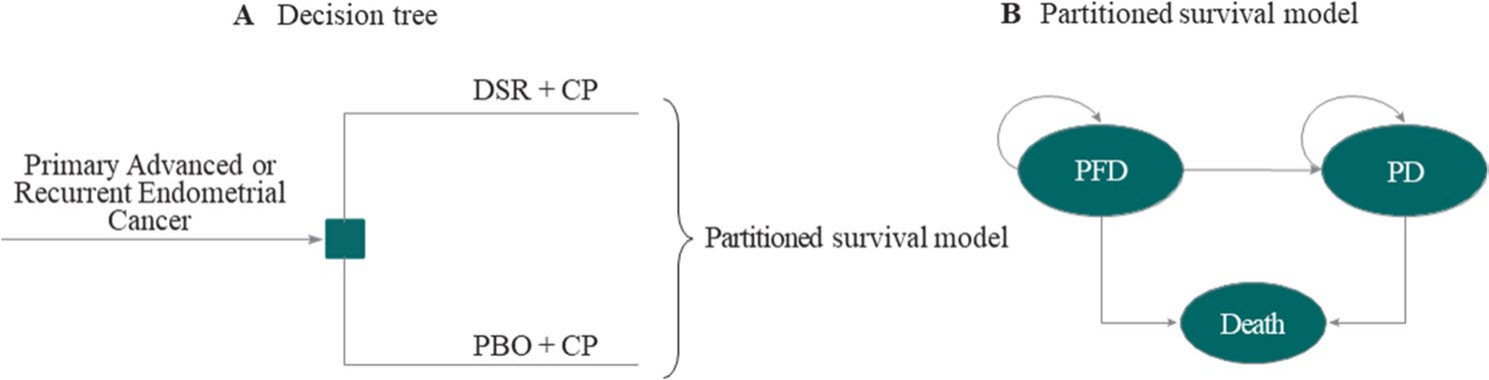
Structure of the hybrid economic model. **A** Decision tree representing the initial treatment choice between DSR + CP and PBO + CP. **B** A partitioned survival model is used to simulate patient prognosis over time. The model comprises three mutually exclusive health states: Progression-Free Disease (PFD), Progressive Disease (PD), and death. Arrows indicate possible transitions between states

A partitioned survival model (PSM) was constructed in TreeAge Pro Healthcare 2025 [17] to evaluate the cost-utility of the intervention from the Saudi healthcare payer’s perspective. The model simulated patient transitions through three mutually exclusive health states: Progression-Free Disease (PFD), Progressive Disease (PD), and death (Fig. 1). All patients entered the model in the PFD state and could transition unidirectionally to PD or Death. The proportion of patients in each state was derived directly from Kaplan-Meier (KM) curves for Progression-Free Survival (PFS) and Overall Survival (OS) published in the RUBY trial. The PFS curve defined PFD state occupancy, whereas PD state occupancy was defined as the difference between the OS and PFS curves. The analysis utilized a 21-day cycle length over a lifetime horizon (ages 64–80), corresponding to the median patient age and local female life expectancy at that time. Adhering to the recommended best practices in health economic evaluations, a 3% annual discount rate was applied to both costs and QALYs [18].

### Target population and interventions

The model simulated a cohort of patients with pA/rEC, with baseline characteristics and inclusion criteria drawn from the overall RUBY trial population [9]. For the analysis, this cohort was partitioned into the overall population, dMMR subgroup, and pMMR subgroup, consistent with trial stratification. The first-line therapy replicated the treatment arms of the RUBY trial. Patients received either DSR (500 mg IV every 3 weeks for six cycles, then 1000 mg IV every 6 weeks) or a matching placebo (PBO). Both were administered concurrent chemotherapy (carboplatin AUC 5 and paclitaxel 175 mg/m² q3w for six cycles). Maintenance therapy with DSR or PBO was continued for up to 3 years or until disease progression or unacceptable toxicity. Following progression to first-line therapy, patients transitioned to subsequent active treatment or best supportive care (BSC). The probability of receiving active subsequent therapy was based on the differential utilization rates observed in the RUBY trial [9]. The selection of subsequent treatment regimens in the model was based on the official, authoritative source for current clinical practice and reimbursement policy for EC in Saudi clinical practice, as well as the local Council of Health Insurance (CHI) EC Treatment Algorithm [19], which incorporates or adapts insights from international authorities [19]. Modeled therapeutic options included pembrolizumab monotherapy for patients with dMMR tumors and either pembrolizumab plus lenvatinib or single-agent chemotherapy (predominantly doxorubicin) for patients with pMMR tumors. A key assumption in the model was that patients in DSR + CP would not receive further immunotherapy. The time for these subsequent treatments was assumed to correspond to the median PFS from key clinical trials, including KEYNOTE-158 for pembrolizumab [20] and KEYNOTE-775 for the lenvatinib combination and doxorubicin [21]. Patients ineligible for or discontinuing active therapy received BSC, which is defined as multidisciplinary palliative care.

### Survival analysis and model parameters

#### Data reconstruction from KM curves in the RUBY trial

In the absence of individual patient data (IPD), time-to-event data for PFS and OS were reconstructed from published KM curves of the RUBY trial. The reconstruction procedure involved two stages: (i) digitization of the graphical curves using WebPlotDigitizer (v5.2) to extract numerical data points [22] and (ii) the application of the Hoyle et al. algorithm to these points to generate a pseudo-IPD dataset suitable for analysis [23].

#### Parametric model fitting

To enable extrapolation of survival outcomes beyond the trial’s follow-up period, a range of standard parametric survival models were systematically fitted to the reconstructed PFS and OS data. The distributions evaluated were the Exponential, Weibull, Gompertz, Log-logistic, Log-normal, and Generalized Gamma Distributions. All models were fitted using the survival, flexsurv, and survminer packages in R [24].

#### Model selection and validation

The optimal parametric model for each endpoint was selected based on a formal goodness-of-fit assessment. This assessment included both statistical and visual criteria. The statistical fit was quantified using the Akaike Information Criterion (AIC) and Bayesian Information Criterion (BIC), where lower values indicate a preferred model (Supplementary Table S1). Statistical selection was corroborated by visual inspection, which compared the fitted parametric curve with the reconstructed KM plot to ensure clinical plausibility (Supplementary Figure S2).

#### Derivation of model parameters

The selected parametric functions were used to derive the key parameters for the economic model. The transition probabilities for PFS and OS were calculated directly from the survival functions. Disease-specific mortality was derived from the extrapolated OS curve and combined with the background mortality rates for the general Saudi Arabian population [25] to estimate OS. The final clinical efficacy inputs are summarized in Table 1.

**Table 1:** Baseline Parameter Values for Clinical Efficacy Models of Survival Outcomes in the RUBY Trial by Population and Treatment Group

Model	Parameter	Baseline Value
PFS - Overall Population		
Weibull (DSR + CP Group)	Scale Parameter (λ)	21.4562
	Shape Parameter (k)	1.0189
LogNormal (PBO + CP Group)	Mean Parameter (µ)	2.2501
	Standard Deviation (σ)	0.9401
OS - Overall Population		
Gompertz (DSR + CP Group)	Shape Parameter (η)	0.0008
	Scale Parameter (b)	0.0153
Weibull (PBO + CP Group)	Scale Parameter (λ)	38.3971
	Shape Parameter (k)	1.3501
PFS - dMMR Subgroup		
Generalized Gamma (DSR + CP Group)	Location Parameter (µ)	3.8011
	Scale Parameter (σ)	1.4753
	Shape Parameter (Q)	0.6576
Log-logistic (PBO + CP Group)	Scale Parameter (α)	8.0441
	Shape Parameter (β)	1.9689
OS - dMMR Subgroup		
Gompertz (DSR + CP Group)	Shape Parameter (η)	0.0001
	Scale Parameter (b)	0.0083
Weibull (PBO + CP Group)	Scale Parameter (λ)	40.0051
	Shape Parameter (k)	1.1549
PFS - pMMR Subgroup		
Generalized Gamma (DSR + CP Group)	Location Parameter (µ)	2.8385
	Scale Parameter (σ)	0.8319
	Shape Parameter (Q)	0.8787
Weibull (PBO + CP Group)	Scale Parameter (λ)	14.2913
	Shape Parameter (k)	1.1681
OS - pMMR Subgroup		
Gompertz (DSR + CP Group)	Shape Parameter (η)	0.0206
	Scale Parameter (b)	0.0131
Weibull (PBO + CP Group)	Scale Parameter (λ)	35.9261
	Shape Parameter (k)	1.3603

Model parameters (e.g., scale, shape) are presented for the best-fitting parametric distributions for each survival outcome and treatment arm within the specified patient population

*Abbreviations: CP* Carboplatin-Paclitaxel, *dMMR* mismatch repair deficient, *DSR* Dostarlimab, *OS* Overall Survival, *PBO* Placebo, *PFS* Progression-Free Survival, *pMMR* mismatch repair proficient

The analysis of adverse events was restricted to severe events (grade 3 or higher), with an incidence of ≥ 5% in the RUBY trial, a common threshold in health economic evaluations [26]. The incidence rates for these selected events were extracted from the trial publications to parameterize the model with associated management costs and quality-of-life decrements (Table 2).

**Table 2:** Incidence of Selected Grade 3 or higher Adverse Events in the RUBY Trial by Treatment Group

AE Incidence	Variable	Baseline Value	Range (Min)	Range (Max)
DSR + CP Group	Anemia	0.149	0.1192	0.1788
	Neutropenia	0.095	0.076	0.114
	Neutrophil count decreased	0.083	0.0664	0.0996
	Lymphocyte count decreased	0.054	0.0432	0.0648
	White-cell count decreased	0.066	0.0528	0.0792
	Hypertension	0.071	0.0568	0.0852
	Pulmonary embolism	0.05	0.04	0.06
	Hypokalemia	0.05	0.04	0.06
PBO + CP Group	Anemia	0.163	0.1304	0.1956
	Neutropenia	0.093	0.0744	0.1116
	Neutrophil count decreased	0.138	0.1104	0.1656
	Lymphocyte count decreased	0.073	0.0584	0.0876
	White-cell count decreased	0.053	0.0424	0.0636
	Hypertension	0.033	0.0264	0.0396
	Pulmonary embolism	0.049	0.0392	0.0588
	Hypokalemia	0.037	0.0296	0.0444

Data are presented as incidence rates (proportions)

*Abbreviations: AE* adverse event, *PBO + CP* placebo plus carboplatin–paclitaxel, *DSR + CP* dostarlimab plus carboplatin–paclitaxel

### The model outcomes

The model adopted a Saudi Arabian base case and considered the direct medical costs from a payer perspective. The primary outcomes of the analysis were QALYs, incremental costs, and ICER, which was defined as the incremental cost per QALY gained. A critical component of this economic evaluation is determining a robust WTP threshold. As Saudi Arabia has not formally legislated a national WTP threshold [27], a threshold must be established based on established methodologies. Based on 2024 data from the General Authority for Statistics (GASTAT), Saudi Arabia’s annual gross domestic product (GDP) per capita is approximately $30,935.

Standard WTP thresholds often fail to adequately reflect societal preferences when evaluating highly specialized oncology therapeutics for severe disease states. To address this, neighboring high-income Gulf Cooperation Council (GCC) countries, such as the United Arab Emirates, have implemented explicit frameworks permitting WTP thresholds of up to three times the GDP per capita for severe and rare conditions [28]. This elevated benchmark is also robustly supported by local Saudi literature. Recent economic evaluations addressing high-burden conditions, such as lung cancer screening [29] and multiple sclerosis [30], have explicitly recommended and utilized a 3x GDP threshold (approximately $99,120). Furthermore, systematic reviews of local economic evaluations confirm the widespread acceptance of this 3x GDP upper limit [31].

In alignment with these regional frameworks, established local precedents, and the upper boundary of the widely cited WHO-CHOICE range (spanning one to three times the GDP per capita), this study adopted a primary WTP threshold of $90,000, representing 2.9x the national GDP per capita. The selection of a value at this higher end is considered justifiable and appropriate for a high-income nation evaluating innovative health technologies [32, 33].

To address the uncertainty associated with this threshold, we performed a probabilistic sensitivity analysis (PSA) and generated Cost-Effectiveness Acceptability Curves (CEACs). CEACs present the probability that the intervention is cost-effective over a wide continuum of WTP values, offering a more complete assessment of economic value than a static ICER comparison [34].

### Data sourcing for cost inputs

An economic evaluation was conducted from the perspective of a healthcare payer within the context of the Saudi Arabian healthcare system. The analysis was limited to direct medical costs, which were standardized to 2025 US dollars (USD). The analysis encompassed drug acquisition and administration, routine patient monitoring, management of severe adverse events (AEs) (grade 3 or higher), subsequent lines of therapy, and best supportive care (BSC). For cost components with official tariffs, data were sourced directly; specifically, the acquisition costs for all therapeutic agents, including index and subsequent therapies, were derived from the official Saudi Food and Drug Authority (SFDA) drug database [35].

For key medical costs for which published local data were unavailable, a formal Structured Expert Elicitation (SEE) was designed and conducted to ensure robust and contextually relevant estimates. This process was specifically employed to quantify the costs of managing grade ≥ 3 AEs with an incidence of 5% or higher in the RUBY trial (e.g., anemia, neutropenia, pulmonary embolism), as well as the costs for routine follow-up care and end-of-life best supportive care. Aligning with ISPOR Task Force Good Practice Guidelines [36], our SEE process mandated that panelists possess a minimum of 10 years of active clinical practice in Saudi oncology or equivalent expertise in health economics. The finalized multidisciplinary panel consisted of 17 Saudi-based experts: 6 board-certified gynecologic oncologists, 2 hematologists, 4 oncology clinical pharmacists, and 5 health economists.

Following a comprehensive briefing and training on three-point estimation techniques and cognitive bias mitigation, a robust two-round modified Delphi approach was implemented via the secure online Qualtrics platform. In the first round, experts independently provided cost estimates (minimum, most likely, maximum). To formally quantify consensus after this initial round, the interquartile range (IQR) and coefficient of variation were calculated for all cost parameters. Estimates falling outside 1.5x IQR were classified as extreme outliers. To actively mitigate cognitive bias, experts who supplied these outliers were required to either submit a documented clinical rationale or independently revise their estimates in the subsequent round.

Ultimately, base-case parameters were aggregated through equal-weight linear pooling, and the resulting three-point estimates were used to parameterize beta-PERT distributions for each component utilizing the Excel-based EXPert eLICItation (EXPLICIT) tool. The derived mean costs then underwent face validity checks with an expert panel to ensure clinical and economic plausibility. These finalized values were directly integrated into the economic model. For the base-case analysis, the mean value from each fitted beta-PERT distribution was applied. The derived IQRs served as the predefined boundaries for the deterministic sensitivity analysis, while the full distributions were sampled for the probabilistic sensitivity analysis (PSA) to robustly characterize the impact of cost uncertainty. A detailed summary of all cost inputs, their mean values, distributions, and specific sources is provided in Table 3.

**Table 3:** Base-case values, Distributions, and Ranges for Model Cost and Utility Inputs

Category	Subcategory	Variable	Baseline Value	Distribution	Range (Min)	Range (Max)	Reference
Cost Parameters (per cycle) ($)	Drug Cost *	Dostarlimab	7,460.19	Gamma	-30%	+ 30	[35]
		Pembrolizumab	3,130.26	Gamma	-30%	+ 30	[35]
		Paclitaxel	5,267.60	Gamma	-30%	+ 30	[35]
		Carboplatin	375.08	Gamma	-30%	+ 30	[35]
		lenvatinib	6,879.19	Gamma	-30%	+ 30	[35]
		doxorubicin	2,197.54	Gamma	-30%	+ 30	[35]
		Best supportive care cost	2,200	Beta-PERT	IQR Q1 = 1,030	IQR Q3 = 3,370	Structured Expert Elicitation
	Healthcare Resource Costs	Routine follow-up	3,843.45	Beta-PERT	IQR Q1 = 2,976.45	IQR Q3 = 4,710.45	Structured Expert Elicitation
	AE Costs	Anemia	1,371.15	Beta-PERT	IQR Q1 = 708.15	IQR Q3 = 2,034.15	Structured Expert Elicitation
		Neutropenia	2,076.54	Beta-PERT	IQR Q1 = 1,569.54	IQR Q3 = 2,583.54	Structured Expert Elicitation
		Neutrophil count decreased	2,176.54	Beta-PERT	IQR Q1 = 1,735.54	IQR Q3 = 2,617.54	Structured Expert Elicitation
		Lymphocyte count decreased	1,275.43	Beta-PERT	IQR Q1 = 900.43	IQR Q3 = 1,650.43	Structured Expert Elicitation
		White-cell count decreased	1,108.02	Beta-PERT	IQR Q1 = 766.02	IQR Q3 = 1,450.02	Structured Expert Elicitation
		Hypertension	688.39	Beta-PERT	IQR Q1 = 226.39	IQR Q3 = 1,150.39	Structured Expert Elicitation
		Pulmonary embolism	897.57	Beta-PERT	IQR Q1 = 762.57	IQR Q3 = 1,032.57	Structured Expert Elicitation
		Hypokalemia	311.09	Beta-PERT	IQR Q1 = 260.09	IQR Q3 = 362.09	Structured Expert Elicitation
Utility Parameters	Health State Utility	PFS	0.817	Beta	0.6536	0.9804	[37]
		PD	0.779	Beta	0.6232	0.9348	[37]
	AEs disutility	Anemia	-0.073	Beta	-0.0584	-0.0876	[38]
		Neutropenia	-0.09	Beta	-0.072	-0.108	[39]
		Neutrophil count decreased	-0.09	Beta	-0.072	-0.108	[39]
		Lymphocyte count decreased	-0.09	Beta	-0.072	-0.108	[39]
		White-cell count decreased	-0.09	Beta	-0.072	-0.108	[39]
		Hypertension	-0.05	Beta	-0.04	-0.06	[39]
		Pulmonary embolism	-0.1	Beta	-0.08	-0.12	Estimated
		Hypokalemia	-0.05	Beta	-0.04	-0.06	Estimated

All costs are presented in 2025 US Dollars ($) from a Saudi payer perspective

Estimated: The values for pulmonary embolism and hypokalemia disutilities were based on clinical plausibility relative to other sourced disutilities and varied extensively in the sensitivity analyses

*Abbreviations: AE* adverse event, *FDA* Food and Drug Authority, *PD* progressive disease, *PFS* progression-free survival

*Patients’ body surface area 1.88 m² [40]

### Utility and QALY calculation

The QALYs were calculated by multiplying the time spent in each model’s health state by its assigned utility value. The quality-of-life impact of severe adverse events was modeled as a one-time disutility applied in the cycle of occurrence. All the utility parameters used in the analysis are presented in Table 3.

### Sensitivity analysis

To thoroughly assess the stability of the base-case results and effectively characterize the decision uncertainty, a two-pronged sensitivity analysis was performed, following the best practice guidelines from the ISPOR-SMDM. First, a series of one-way deterministic sensitivity analyses (DSA) was performed to identify the key drivers of the model’s outcomes. This involved systematically varying each Parameter across a plausible range using 95% confidence intervals where available. In cases where empirical data for specific parameters are lacking, a conservative standardized range is applied: ±20% for probabilities and utilities and ± 30% for costs. This approach is common and accepted in health economic modeling, ensuring that all influential parameters are examined.

Although DSA is essential for pinpointing key parameters, it does not account for the combined effects of simultaneous uncertainties. Therefore, PSA was essential for evaluating overall confidence in the cost-effectiveness conclusion. This was accomplished using a second-order Monte Carlo simulation of 1,000 iterations, where all parameters were simultaneously sampled from their respective probability distributions (Table 4). The findings are illustrated using CEACs and cost-effectiveness scatterplots.

**Table 4:** Base-Case Cost-Effectiveness Analysis by Mismatch Repair (MMR) Status

Group		Costs ($*)	ΔCosts ($*)	QALYs	ΔQALYs	ICER ($*/QALY)
Overall	PBO + CP	164,702	-	3.07	-	-
	DSR + CP	309,419	144,716	4.62	1.55	93,243.88
dMMR	PBO + CP	173,532	-	2.24	-	-
	DSR + CP	518,836	345,303	6.42	4.17	82,754.46
pMMR	PBO + CP	154,962	-	2.70	-	-
	DSR + CP	232,964	78,002	3.29	0.59	132,571.11

*Abbreviations: CP* chemotherapy, *dMMR* deficient mismatch repair, *pMMR* proficient mismatch repair, *DSR* dostarlimab, *ICER* incremental cost-effectiveness ratio, *PBO* placebo, *QALY* quality-adjusted life year

*Costs reported in 2025 US dollars

## Results

### Base-case analysis

The results are presented in Table 4. In the overall population, the DSR + CP group demonstrated an incremental effect of 1.55 QALYs and an incremental cost of $144,716, resulting in an ICER of $93,243.88 per QALY compared to the PBO + CP group. In the dMMR and pMMR subgroups, DSR + CP incurred incremental costs of $345,303 and $78,002, respectively, compared to PBO + CP. This resulted in incremental effects of 4.17 QALYs and 0.59 QALYs, yielding ICERs of $82,754.46/QALY and $132,571.10/QALY, respectively. With a WTP threshold of $90,000 per QALY, DSR + CP is a cost-effective first-line treatment for advanced EC relative to PBO + CP in the dMMR subgroup but not in the pMMR subgroup.

### Sensitivity analysis

**Fig. 2:**
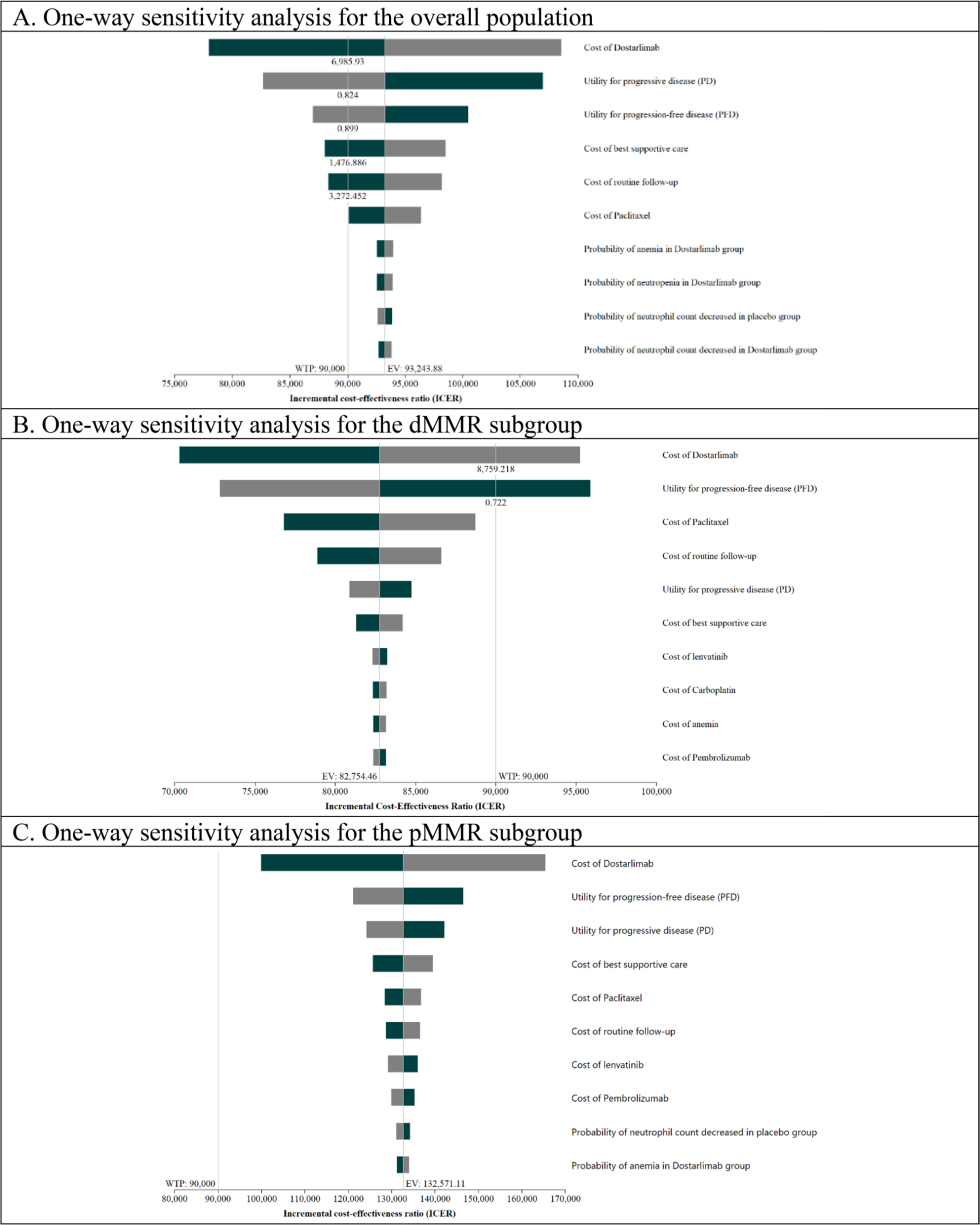
Deterministic sensitivity analysis outputs: Tornado diagram

The results from DSA, depicted in the tornado diagram (Fig. 2), pinpointed the cost of DSR + CP as the primary driver of the ICER. The analysis demonstrated that the likelihood of the ICER exceeding the $90,000 threshold was markedly sensitive to variations in the DSR + CP costs across all examined subgroups. The utilities associated with PD and PFD also showed a notable impact; the uncertainty surrounding these utility values had the potential to shift the ICER across the WTP threshold, a factor particularly evident in the overall population.

**Fig. 3:**
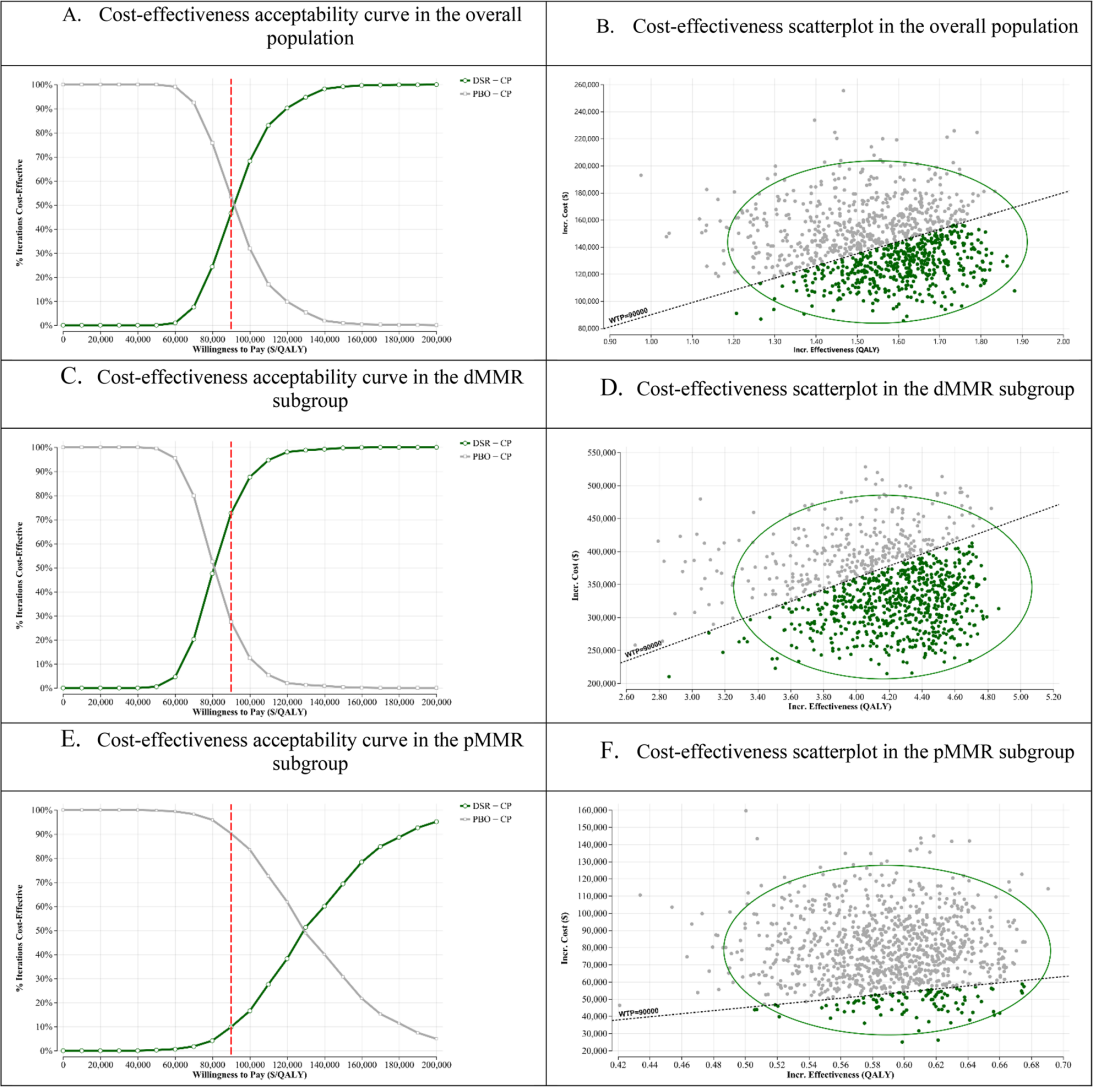
Probabilistic sensitivity analysis outputs

Figure 3 illustrates the findings derived from PSA. The CEAC for the overall population indicates that an increase in the WTP threshold correlates with a higher probability of DSR + CP not being deemed cost-effective (Fig. 3A). At the designated WTP threshold of $90,000 (represented by the vertical dashed red line), DSR + CP exhibits a notably lower probability (46.5%) of being a cost-effective alternative than PBO + CP (53.5%). The scatterplot illustrating incremental costs against incremental effectiveness for DSR + CP compared to PBO + CP within the overall population revealed a broad distribution across 1,000 simulated iterations (Fig. 3B). A significant number of data points (dark green) are situated over the WTP threshold line of $90,000. A significant concentration of points in the northeast quadrant indicates that DSR + CP is more effective, albeit at a higher cost. The ellipse includes most of these simulations and provides a visual representation of the uncertainty surrounding the mean ICER.

Within the dMMR subgroup, CEAC indicates a significantly greater likelihood of DSR + CP being cost-effective at all WTP thresholds when compared to the overall population (Fig. 3C). At the $90,000 WTP threshold, DSR + CP demonstrated a significant likelihood (72.7%) of being cost-effective, whereas PBO + CP exhibited a notably lower probability (27.4%). The scatterplot for the dMMR subgroup indicates that a significant proportion of the simulated ICERs are situated below the WTP threshold of $90,000 (Fig. 3D). The distribution of points is primarily concentrated in the northeast quadrant, suggesting a correlation between higher effectiveness and increased cost for DSR + CP while also demonstrating a significant inclination towards cost-effectiveness at typical WTP values.

In the pMMR subgroup, CEAC demonstrated a reduced likelihood of DSR + CP being cost-effective when compared to the overall population and dMMR subgroup (Fig. 3E). At the $90,000 WTP threshold, DSR + CP demonstrates a lower likelihood (9.8%) of being cost-effective, whereas DSR + CP exhibits a higher likelihood (90.2%). DSR + CP demonstrates a higher likelihood of cost-effectiveness only at WTP thresholds considerably greater than $132,571.11. The scatterplot for the pMMR subgroup indicates that a small number of simulated ICERs (dark green) were positioned below the WTP threshold line of $90,000 (Fig. 3F).

## Discussion

Our base-case analysis, when evaluated against the WTP threshold used in this study, revealed a clear divergence in value across patient populations. The addition of DSR was cost-effective for the dMMR subgroup, with their respective ICERs falling below the specified threshold. In stark contrast, the therapy was unequivocally not cost-effective for the pMMR subgroup, as its ICER significantly exceeded the WTP threshold.

This pattern of differential cost-effectiveness based on biomarker status is remarkably consistent with international findings, although specific economic outcomes vary in ways that highlight the unique context of our analysis. For instance, studies in the US found DSR + CP to be cost-effective for the dMMR population, reporting even more favorable ICERs [10, 11]. Conversely, analyses in Canada and China also concluded that therapy was cost-effective for the dMMR subgroup but reported much lower ICERs [12, 13]. A primary cause of these differences is the significant variation in national healthcare costs and pricing structures. Our model’s cost inputs, derived from a rigorous Structured Expert Elicitation process, are tailored to the Saudi system and thus differ from the database costs used in other countries.

Furthermore, the ultimate conclusion of “cost-effectiveness” is always relative to a country’s WTP threshold, which reflects broader national economic and healthcare priorities. Despite variations between countries, our DSA strongly supports the universal conclusion that the acquisition cost of DSR + CP is undeniably the most significant factor influencing the ICER. These findings highlight that the primary obstacle to the value proposition of therapy is its price, a critical insight for subsequent policy implications.

The results of this economic evaluation provide direct, evidence-based implications for Saudi healthcare. The most immediate clinical implication is the necessity to mandate dMMR/pMMR biomarker testing as a prerequisite for therapy, which is strongly supported by updated National Comprehensive Cancer Network (NCCN) guidelines [41]. Our analysis provides a clear economic justification for this “test-all” strategy by showing that therapy is not cost-effective in the pMMR population, even with a generous WTP threshold. Providing DSR + CP without biomarker confirmation would result in a significant expenditure of resources for a benefit that does not justify the cost. A test-all policy is therefore essential to target this high-cost therapy exclusively to the patient population that stands to gain the most, preventing needless financial toxicity for the healthcare system [42].

From a policy perspective, our findings provide a clear mandate for national procurement bodies, such as the National Unified Procurement Company (NUPCO), the exclusive, state-owned entity responsible for centralized procurement and supply chain management of pharmaceuticals and medical supplies for SA’s governmental healthcare sector, and regulatory authorities. The DSA unequivocally identified the drug price as the primary driver of the ICER, meaning that reimbursement at the current price is not economically viable, particularly for the pMMR group. This evidence empowers Saudi negotiators to pursue aggressive price reductions, even for the dMMR subgroup, where therapy was technically cost-effective in our model. The goal should be to further enhance the value proposition of therapy.

Based on these clinical and economic realities, a stratified reimbursement policy is the most rational approach. For patients with dMMR EC, reimbursement for DSR + CP should be granted but made conditional upon the successful negotiation of a value-based price [43]. Conversely, for the pMMR population, reimbursement should be denied at current international price levels owing to the combination of unfavorable clinical efficacy as a monotherapy and a lack of cost-effectiveness when used in combination with chemotherapy in the Saudi context. NUPCO and other payers should proactively develop and utilize Managed Entry Agreements (MEAs) to manage the budgetary impact further and mitigate the financial risk associated with this high-cost therapy. Initially, these could take the form of financial-based agreements, such as discounts or price-volume arrangements, with a long-term vision of building capacity for more complex outcome-based contracts [44].

Several limitations that highlight the methodological rigor of this study must be considered. A primary limitation is the absence of a formally established WTP threshold for the Saudi economic landscape; however, we addressed this by presenting CEACs to show the probability of cost-effectiveness across a range of thresholds [45]. A second notable limitation is our reliance on non-Saudi utility value sets. Recent validations of the EQ-5D-5L for the Saudi population demonstrate higher sensitivity and greater disutility decrements in the pain and anxiety dimensions when compared to Western norms [46]. Consequently, applying these external weights may underestimate the severe physical and psychological burden associated with the ‘Progressive Disease’ state among Saudi women. While we mitigated this uncertainty by robustly varying these values in our sensitivity analysis, future evaluations should integrate localized value sets to more accurately capture these health-state utilities. Finally, to address the common challenge of the unavailability of published local cost data for key medical events, we employed a formal SEE. This is a valid and internationally recognized scientific method for generating robust parameter estimates in the absence of empirical data [36].

## Conclusion

In conclusion, this economic evaluation demonstrates that, at its current modeled price, the addition of DSR to CP is not a cost-effective strategy for pA/rEC patients with pMMR status and presents a questionable value proposition, even for the dMMR subgroup within the Saudi context. The therapy’s high incremental cost is the primary barrier to its adoption. To integrate this innovation responsibly, healthcare policy should require biomarker testing to limit its use to the dMMR population. Additionally, reimbursement should be contingent on significant price reductions. Future research should focus on developing a new Saudi-specific economic model and conducting a comprehensive Budget Impact Analysis. These steps are crucial to ensure that advancements in cancer care align with the Kingdom’s strategic goals.

## Supplementary Information

Supplementary Material 1.

## Data Availability

The data that support the findings of this study are available from the corresponding author, ZSA, upon reasonable request.

## Abbreviations

AE: Adverse event
PBO + CP: Placebo plus carboplatin–paclitaxel
DSR + CP: Dostarlimab plus carboplatin–paclitaxel
