# Supplementary material for: Cost-utility of first-line dostarlimab plus carboplatin-paclitaxel compared with placebo for patients with pA/rEC: a partitioned survival analysis
Source: Health Econ Rev. 2026 Apr 23;16:71. doi: 10.1186/s13561-026-00778-0 (PMC13237884; doi:10.1186/s13561-026-00778-0)
Supplement: Supplementary file 1 — Supplementary Material 1. [file 13561_2026_778_MOESM1_ESM.docx]

**Supplementary Material**

**Cost-Utility of First-Line Dostarlimab Plus Carboplatin-Paclitaxel Compared with Placebo for Patients with pA/rEC: A Partitioned Survival Analysis**

Ziyad S Almalki^a*^, Saba S Albanna^b^, Yahia A Hadadi^c^, Ahmed M Alshehri^a^ , Kamar Z Jamal^b^, Saja H Almazrou^d^, Jameilh A Alsamiri^b^, Abdulrahman A Alsuhibani^e^, Abdullah A Alalwan^a^, Ahmad A Alamer^a^, Nehad J Ahmed^a^, Abeer A Shhada^b^, Ghuafran A Alnajem^b^

^a^Department of Clinical Pharmacy, College of Pharmacy, Prince Sattam Bin Abdulaziz University, Al-Kharj, Riyadh, Saudi Arabia; ^b^ Department of Clinical Pharmacy, College of Pharmacy, AlMaarefa University, Riyadh, Saudi Arabia; ^c^ Department of Sales, A. Menarini Farmaceutica Internazionale S.R.L, Riyadh, Saudi Arabia; ^d^ Department of Clinical Pharmacy, College of Pharmacy, King Saud University, Riyadh, Saudi Arabia; ^e^ Department of Pharmacy Practice, College of Pharmacy, Qassim University, Qassim, Saudi Arabia.

Correspondence: Ziyad Saeed Almalki, Department of Clinical Pharmacy, College of Pharmacy, Prince Sattam Bin Abdulaziz University, Al-Kharj, Riyadh, Saudi Arabia

Tel (+966) 11 588 7315

Email z.almalki@psau.edu.sa

**Table S1.** Goodness-of-Fit Statistics for Parametric Survival Models.

| **Population Group** | **Distribution** | **DSR + CP (AIC)** | **DSR + CP (BIC)** | **PBO + CP (AIC)** | **PBO + CP (BIC)** |
| --- | --- | --- | --- | --- | --- |
| **OS-Overall Population** | Exponential | 625.83 | 628.31 | 718.49 | 721.17 |
|  | Weibull | 607.22 | 612.17 | **705.71** | **711.05** |
|  | Gompertz | **601.29** | **606.24** | 706.17 | 711.52 |
|  | logistic | 620.51 | 625.46 | 726.27 | 731.62 |
|  | Log-normal | 628.92 | 633.88 | 724.62 | 729.96 |
|  | Log-logistic | 622.71 | 627.66 | 714.12 | 719.46 |
|  | Generalized Gamma | 601.71 | 609.14 | 706.83 | 714.85 |
| **OS-dMMR** | Exponential | 322.74 | 325.21 | 507.85 | 510.41 |
|  | Weibull | 322.78 | 327.69 | **499.49** | **504.57** |
|  | Gompertz | **319.91** | **324.82** | 502.26 | 507.35 |
|  | logistic | 334.93 | 339.84 | 521.01 | 526.11 |
|  | Log-normal | 327.87 | 332.78 | 502.24 | 507.33 |
|  | Log-logistic | 326.04 | 330.95 | 501.41 | 506.49 |
|  | Generalized Gamma | 322.01 | 329.36 | 501.38 | 509.01 |
| **OS-pMMR** | Exponential | 597.17 | 599.63 | 682.73 | 685.37 |
|  | Weibull | 572.96 | 577.87 | **675.34** | **680.61** |
|  | Gompertz | **570.84** | **575.74** | 676.08 | 681.35 |
|  | logistic | 586.46 | 591.37 | 699.31 | 704.57 |
|  | Log-normal | 589.18 | 594.09 | 693.42 | 698.69 |
|  | Log-logistic | 586.02 | 590.92 | 681.73 | 687.01 |
|  | Generalized Gamma | 571.63 | 578.99 | 676.83 | 684.73 |
| **PFS-Overall Population** | Exponential | 752.39 | 755.18 | 762.61 | 765.52 |
|  | Weibull | **740.82** | **746.41** | 762.13 | 767.94 |
|  | Gompertz | 741.51 | 747.11 | 764.45 | 770.26 |
|  | logistic | 776.39 | 781.99 | 820.38 | 826.19 |
|  | Log-normal | 744.91 | 750.49 | **757.01** | **762.81** |
|  | Log-logistic | 746.36 | 751.96 | 758.11 | 763.91 |
|  | Generalized Gamma | 742.82 | 751.21 | 758.95 | 767.67 |
| **PFS-dMMR** | Exponential | 340.75 | 343.39 | 575.63 | 578.47 |
|  | Weibull | 342.07 | 347.35 | 577.61 | 583.31 |
|  | Gompertz | 336.34 | 341.63 | 574.21 | 579.89 |
|  | logistic | 386.22 | 391.51 | 641.71 | 647.39 |
|  | Log-normal | 335.98 | 341.27 | 572.23 | 577.92 |
|  | Log-logistic | 339.21 | 344.49 | **571.59** | **577.28** |
|  | Generalized Gamma | **333.11** | **341.05** | 574.08 | 582.61 |
| **PFS-pMMR** | Exponential | 759.13 | 761.94 | 800.83 | 803.75 |
|  | Weibull | 748.31 | 753.91 | **798.02** | **802.86** |
|  | Gompertz | 745.56 | 751.16 | 802.21 | 808.04 |
|  | logistic | 780.11 | 785.72 | 859.45 | 865.28 |
|  | Log-normal | 758.92 | 764.52 | 807.93 | 813.77 |
|  | Log-logistic | 758.11 | 763.72 | 801.12 | 806.96 |
|  | Generalized Gamma | **739.59** | **748.01** | 802.51 | 811.26 |

Lower Akaike information criterion (AIC) and Bayesian information criterion (BIC) values indicate a better model fit.

Abbreviations: AIC, Akaike information criterion; BIC, Bayesian information criterion; DSR + CP, dostarlimab plus carboplatin-paclitaxel; PBO + CP, placebo plus carboplatin-paclitaxel; OS, overall survival; PFS, progression-free survival; dMMR, mismatch repair deficient; pMMR, mismatch repair proficient.

**Figure S2.** Kaplan-Meier Survival Curves and Parametric Model Fits for the DSR + CP and PBO + CP Cohorts.

| **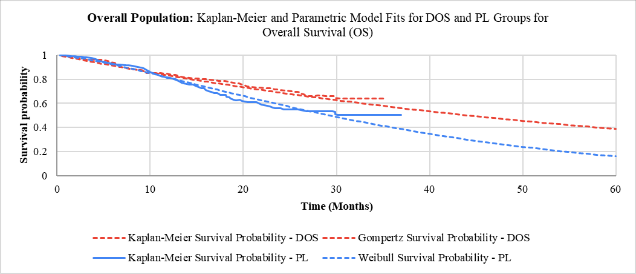** | **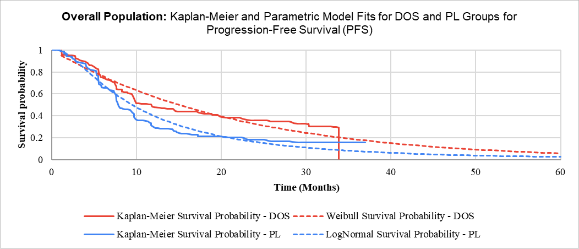** |
| --- | --- |
| **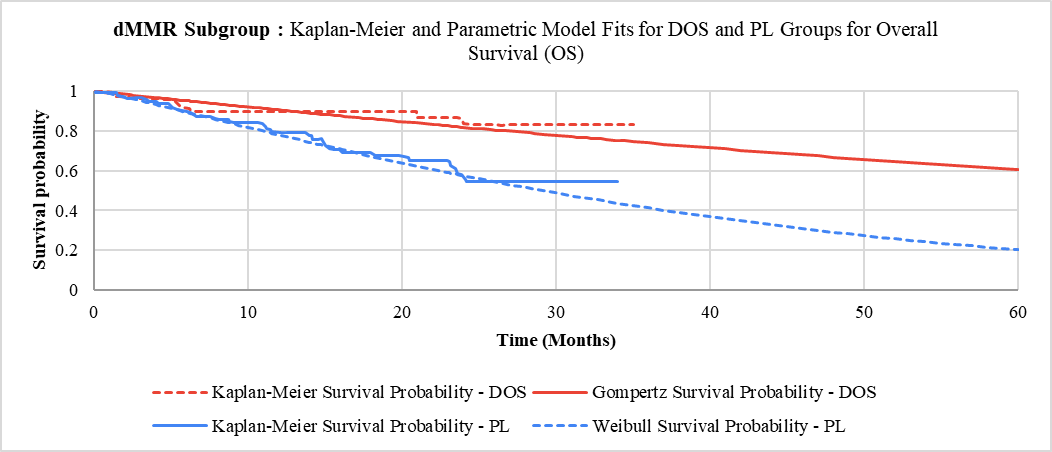** | **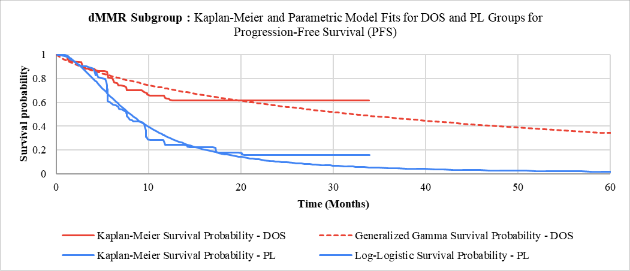** |
| **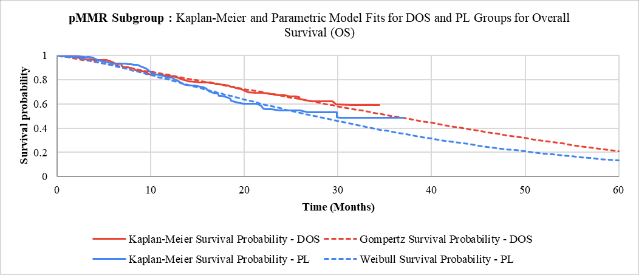** | **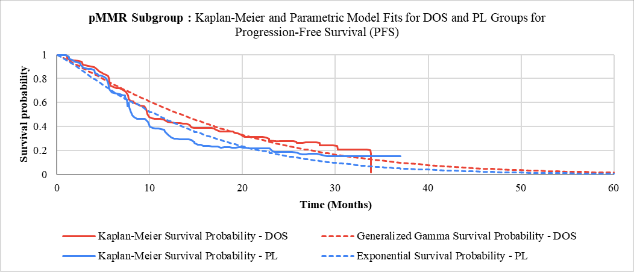** |

The figure presents survival analyses comparing non-parametric Kaplan-Meier estimates (solid lines) with various fitted parametric models (dashed lines). The analyses are shown for the two treatment groups, DSR + CP (red lines) and PBO + CP (blue lines), across different clinical endpoints and patient populations. The y-axis represents the survival probability, and the x-axis represents the time in months. Abbreviations: DSR + CP, dostarlimab plus carboplatin-paclitaxel; PBO + CP, placebo plus carboplatin-paclitaxel; OS, Overall Survival; PFS, Progression-Free Survival; dMMR, deficient Mismatch Repair; pMMR, mismatch repair proficient.

**Table S1.** CHEERS 2022 Checklist.

| **Topic** | **No.** | **Item** | **Reported** |
| --- | --- | --- | --- |
| **Title** |  |  |  |
|  | 1 | Identify the study as an economic evaluation and specify the interventions being compared. | Yes |
| **Abstract** |  |  |  |
|  | 2 | Provide a structured summary that highlights context, key methods, results, and alternative analyses. | Yes |
| **Introduction** |  |  |  |
| **Background and objectives** | 3 | Give the context for the study, the study question, and its practical relevance for decision making in policy or practice. | Yes |
| **Methods** |  |  |  |
| **Health economic analysis plan** | 4 | Indicate whether a health economic analysis plan was developed and where available. | Yes |
| **Study population** | 5 | Describe characteristics of the study population (such as age range, demographics, socioeconomic, or clinical characteristics). | Yes |
| **Setting and location** | 6 | Provide relevant contextual information that may influence findings. | Yes |
| **Comparators** | 7 | Describe the interventions or strategies being compared and why chosen. | Yes |
| **Perspective** | 8 | State the perspective(s) adopted by the study and why chosen. | Yes |
| **Time horizon** | 9 | State the time horizon for the study and why appropriate. | Yes |
| **Discount rate** | 10 | Report the discount rate(s) and reason chosen. | Yes |
| **Selection of outcomes** | 11 | Describe what outcomes were used as the measure(s) of benefit(s) and harm(s). | Yes |
| **Measurement of outcomes** | 12 | Describe how outcomes used to capture benefit(s) and harm(s) were measured. | Yes |
| **Valuation of outcomes** | 13 | Describe the population and methods used to measure and value outcomes. | Yes |
| **Measurement and valuation of resources and costs** | 14 | Describe how costs were valued. | Yes |
| **Currency, price date, and conversion** | 15 | Report the dates of the estimated resource quantities and unit costs, plus the currency and year of conversion. | Yes |
| **Rationale and description of model** | 16 | If modelling is used, describe in detail and why used. Report if the model is publicly available and where it can be accessed. | Yes |
| **Analytics and assumptions** | 17 | Describe any methods for analysing or statistically transforming data, any extrapolation methods, and approaches for validating any model used. | Yes |
| **Characterising heterogeneity** | 18 | Describe any methods used for estimating how the results of the study vary for subgroups. | Yes |
| **Characterising distributional effects** | 19 | Describe how impacts are distributed across different individuals or adjustments made to reflect priority populations. | Yes |
| **Characterising uncertainty** | 20 | Describe methods to characterise any sources of uncertainty in the analysis. | Yes |
| **Approach to engagement with patients and others affected by the study** | 21 | Describe any approaches to engage patients or service recipients, the general public, communities, or stakeholders (such as clinicians or payers) in the design of the study. | Yes |
| **Results** |  |  |  |
| **Study parameters** | 22 | Report all analytic inputs (such as values, ranges, references) including uncertainty or distributional assumptions. | Yes |
| **Summary of main results** | 23 | Report the mean values for the main categories of costs and outcomes of interest and summarise them in the most appropriate overall measure. | Yes |
| **Effect of uncertainty** | 24 | Describe how uncertainty about analytic judgments, inputs, or projections affect findings. Report the effect of choice of discount rate and time horizon, if applicable. | Yes |
| **Effect of engagement with patients and others affected by the study** | 25 | Report on any difference patient/service recipient, general public, community, or stakeholder involvement made to the approach or findings of the study | Yes |
| **Discussion** |  |  |  |
| **Study findings, limitations, generalisability, and current knowledge** | 26 | Report key findings, limitations, ethical or equity considerations not captured, and how these could affect patients, policy, or practice. | Yes |
| **Other relevant information** |  |  |  |
| **Source of funding** | 27 | Describe how the study was funded and any role of the funder in the identification, design, conduct, and reporting of the analysis | Yes |
| **Conflicts of interest** | 28 | Report authors conflicts of interest according to journal or International Committee of Medical Journal Editors requirements. | Yes |

**Table S2.** Akaike information criterion and Bayesian information criterion statistics for alternate parametric survival distributions.

| **Population Group** | **Distribution** | **DOS (AIC)** | **DOS (BIC)** | **PL (AIC)** | **PL (BIC)** |
| --- | --- | --- | --- | --- | --- |
| **OS-Overall Population** | Exponential | 625.83 | 628.31 | 718.49 | 721.17 |
|  | Weibull | 607.22 | 612.17 | **705.71** | **711.05** |
|  | Gompertz | **601.29** | **606.24** | 706.17 | 711.52 |
|  | logistic | 620.51 | 625.46 | 726.27 | 731.62 |
|  | Log-normal | 628.92 | 633.88 | 724.62 | 729.96 |
|  | Log-logistic | 622.71 | 627.66 | 714.12 | 719.46 |
|  | Generalized Gamma | 601.71 | 609.14 | 706.83 | 714.85 |
| **OS-dMMR** | Exponential | 322.74 | 325.21 | 507.85 | 510.41 |
|  | Weibull | 322.78 | 327.69 | **499.49** | **504.57** |
|  | Gompertz | **319.91** | **324.82** | 502.26 | 507.35 |
|  | logistic | 334.93 | 339.84 | 521.01 | 526.11 |
|  | Log-normal | 327.87 | 332.78 | 502.24 | 507.33 |
|  | Log-logistic | 326.04 | 330.95 | 501.41 | 506.49 |
|  | Generalized Gamma | 322.01 | 329.36 | 501.38 | 509.01 |
| **OS-pMMR** | Exponential | 597.17 | 599.63 | 682.73 | 685.37 |
|  | Weibull | 572.96 | 577.87 | **675.34** | **680.61** |
|  | Gompertz | **570.84** | **575.74** | 676.08 | 681.35 |
|  | logistic | 586.46 | 591.37 | 699.31 | 704.57 |
|  | Log-normal | 589.18 | 594.09 | 693.42 | 698.69 |
|  | Log-logistic | 586.02 | 590.92 | 681.73 | 687.01 |
|  | Generalized Gamma | 571.63 | 578.99 | 676.83 | 684.73 |
| **PFS-Overall Population** | Exponential | 752.39 | 755.18 | 762.61 | 765.52 |
|  | Weibull | **740.82** | **746.41** | 762.13 | 767.94 |
|  | Gompertz | 741.51 | 747.11 | 764.45 | 770.26 |
|  | logistic | 776.39 | 781.99 | 820.38 | 826.19 |
|  | Log-normal | 744.91 | 750.49 | **757.01** | **762.81** |
|  | Log-logistic | 746.36 | 751.96 | 758.11 | 763.91 |
|  | Generalized Gamma | 742.82 | 751.21 | 758.95 | 767.67 |
| **PFS-dMMR** | Exponential | 340.75 | 343.39 | 575.63 | 578.47 |
|  | Weibull | 342.07 | 347.35 | 577.61 | 583.31 |
|  | Gompertz | 336.34 | 341.63 | 574.21 | 579.89 |
|  | logistic | 386.22 | 391.51 | 641.71 | 647.39 |
|  | Log-normal | 335.98 | 341.27 | 572.23 | 577.92 |
|  | Log-logistic | 339.21 | 344.49 | **571.59** | **577.28** |
|  | Generalized Gamma | **333.11** | **341.05** | 574.08 | 582.61 |
| **PFS-pMMR** | Exponential | 759.13 | 761.94 | 800.83 | 803.75 |
|  | Weibull | 748.31 | 753.91 | **798.02** | **802.86** |
|  | Gompertz | 745.56 | 751.16 | 802.21 | 808.04 |
|  | logistic | 780.11 | 785.72 | 859.45 | 865.28 |
|  | Log-normal | 758.92 | 764.52 | 807.93 | 813.77 |
|  | Log-logistic | 758.11 | 763.72 | 801.12 | 806.96 |
|  | Generalized Gamma | **739.59** | **748.01** | 802.51 | 811.26 |

This table presents a statistical comparison of seven different parametric distributions used to model survival data. The goal is to determine the best-fitting model for different patient populations and treatment arms.

Endpoints Analyzed: Overall Survival (OS) and Progression-Free Survival (PFS).

Patient Populations: The analysis is performed on the "Overall Population" and two subgroups based on mismatch repair status: "dMMR" (deficient) and "pMMR" (proficient).

Treatment Arms: Two treatment groups are compared, labeled "DOS" and "PL".

Goodness-of-Fit Criteria: The Akaike Information Criterion (AIC) and Bayesian Information Criterion (BIC) are used to evaluate how well each statistical distribution (e.g., Weibull, Gompertz) fits the observed data. For both AIC and BIC, a lower value indicates a better model fit.

Key Findings: Best-Fitting Models

Below are the distributions that provide the best fit for each scenario, based on having the lowest AIC and BIC values.

For Overall Survival (OS)

Overall Population:

DOS Arm: The Gompertz distribution provides the best fit (AIC: 601.29, BIC: 606.24).

PL Arm: The Weibull distribution provides the best fit (AIC: 705.71, BIC: 711.05).

dMMR Subgroup:

DOS Arm: The Gompertz distribution provides the best fit (AIC: 319.91, BIC: 324.82).

PL Arm: The Weibull distribution provides the best fit (AIC: 499.49, BIC: 504.57).

pMMR Subgroup:

DOS Arm: The Gompertz distribution provides the best fit (AIC: 570.84, BIC: 575.74).

PL Arm: The Weibull distribution provides the best fit (AIC: 675.34, BIC: 680.61).

For Progression-Free Survival (PFS)

Overall Population:

DOS Arm: The Weibull distribution provides the best fit (AIC: 740.82, BIC: 746.41).

PL Arm: The Log-normal distribution provides the best fit (AIC: 757.01, BIC: 762.81).

dMMR Subgroup:

DOS Arm: The Generalized Gamma distribution provides the best fit (AIC: 333.11).

PL Arm: The Log-logistic distribution provides the best fit (AIC: 571.59, BIC: 577.28).

pMMR Subgroup:

DOS Arm: The Generalized Gamma distribution provides the best fit (AIC: 739.59, BIC: 748.01).

PL Arm: The Weibull distribution provides the best fit (AIC: 798.02, BIC: 802.86).

**Figure S1.** Kaplan-Meier Survival Curves and Parametric Model Fits for the DOS and PL Cohorts.

| **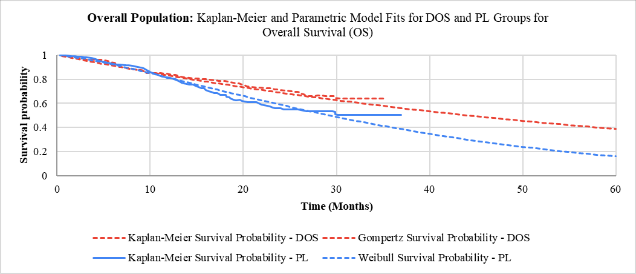** | **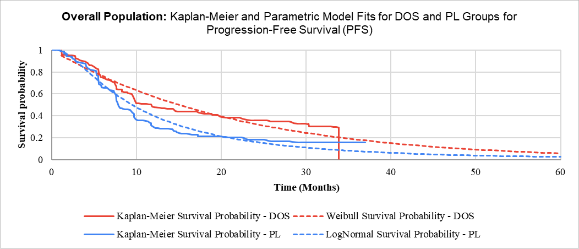** |
| --- | --- |
| **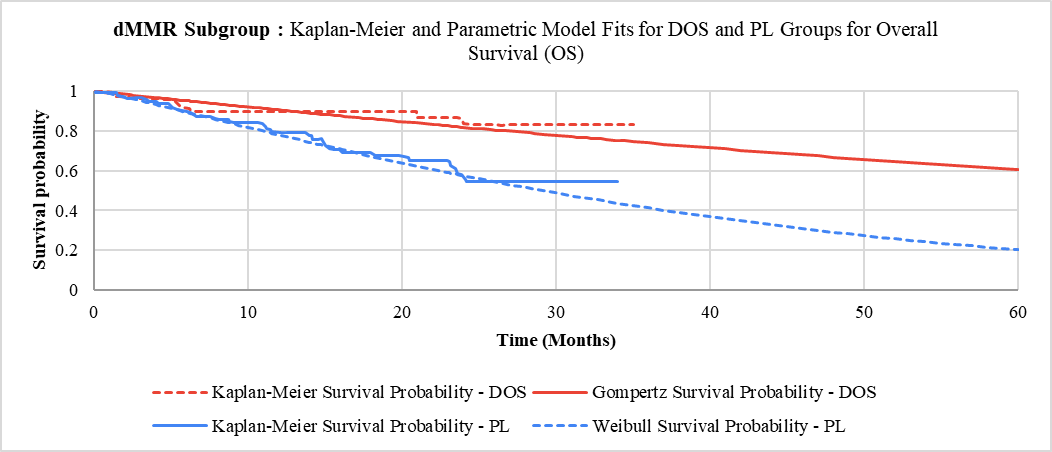** | **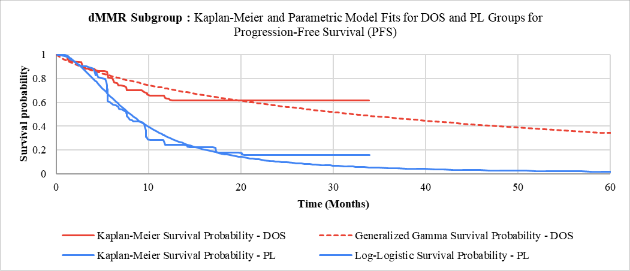** |
| **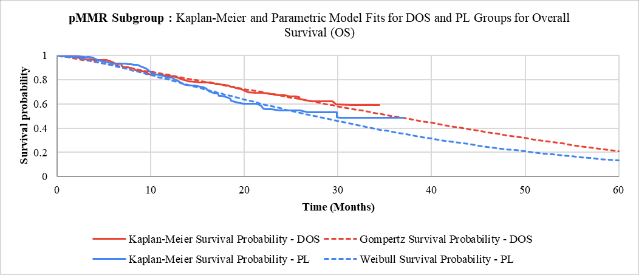** | **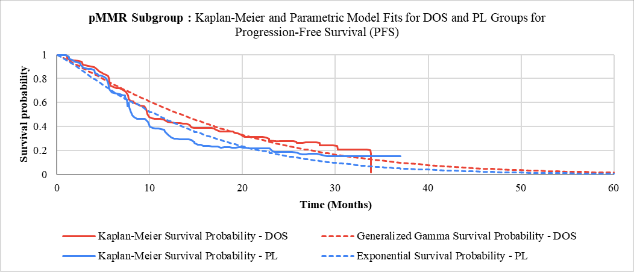** |

The figure presents survival analyses comparing non-parametric Kaplan-Meier estimates (solid lines) with various fitted parametric models (dashed lines). The analyses are shown for two treatment groups, DOS (red lines) and PL (blue lines), across different clinical endpoints and patient populations. the y-axis represents survival probability and the x-axis represents time in months. Abbreviations: DOS, dostarlimab plus carboplatin-paclitaxel; PL, placebo plus carboplatin-paclitaxel; OS, Overall Survival; PFS, Progression-Free Survival; dMMR, deficient Mismatch Repair; pMMR, mismatch repair-proficient.
